# Serosurvey of *Coxiella burnetii* in Police Officers and Working Dogs in Brazil: Case Report and One Health Implications

**DOI:** 10.3390/tropicalmed9040078

**Published:** 2024-04-06

**Authors:** Danilo Alves de França, Jéssica Santos da Silva, Nássarah Jabur Lot Rodrigues, Ana Íris de Lima Duré, João Henrique Farinhas, Louise Bach Kmetiuk, Helio Langoni, Alexander Welker Biondo

**Affiliations:** 1 Department of Animal Production and Preventive Veterinary Medicine, School of Veterinary Medicine and Animals Science, São Paulo State University, Botucatu 05508-220, Brazil; danilo.franca@unesp.br (D.A.d.F.); nassarah.lot@unesp.br (N.J.L.R.); helio.langoni@unesp.br (H.L.); 2 Department of Veterinary Medicine, Federal University of Paraná State, Curitiba 81530-000, Brazil; jessica.silva@ufpr.br (J.S.d.S.); joao.farinhas@ufpr.br (J.H.F.); 3 Service of Virology and Rickettsiosis, Octavio Magalhaes Institute, Ezequiel Dias Foundation, Belo Horizonte 30510-010, Brazil; ana.dure@funed.mg.gov.br; 4 Zoonoses Surveillance Unit, Municipal Secretary of Health, Curitiba 81265-320, Brazil; louisebachk@gmail.com

**Keywords:** One Health, public health, Q fever, zoonoses, serosurvey

## Abstract

Background: Although the *Coxiella burnetii* infection has been investigated in dogs, its role in human transmission remains to be fully established, particularly in close and daily human–dog contact settings, such as in Police K-9 Units. Methods: Accordingly, this study aimed to assess anti-*C. burnetii* antibodies in clinically healthy police officers by an in-house indirect immunofluorescence assay (IFA), and working dogs by a commercial IFA Kit, from the State Special Operations Battalion, Paraná, Southern Brazil. Results: Overall, 1/18 (5.5%) police officers and 9/30 (30.0%; CI 95% 16.66–47.88) dogs tested seropositive to anti-*C. burnetii* IgG antibodies. Conclusions: To date, this is the highest prevalence of Q fever seropositivity among military dogs worldwide. Despite the low sampling rate, a statistically significant association was found between seropositivity and female dogs (*p* = 0.0492). Further studies with larger sample sizes should be conducted to establish the prevalence of Q Fever in other Brazilian K-9 Units. In summary, this study is the first to conduct a concomitant serosurvey of police officers and working dogs, and its findings should be considered a warning for cross-exposure and transmission of *Coxiella burnetii* among Police K-9 Units in Brazil and worldwide.

## 1. Introduction

Despite recent reports throughout Brazil of *Coxiella burnetii* infections in animals and people [1], Q fever is still considered an undetermined disease in the country. Large livestock herds nationwide and in neighboring countries may be the cause of the high prevalence rates observed in recent human serosurveys in Brazil [2,3]. However, infected dogs may also be a potential source of human and animal infections, particularly by eliminating *C. burnetii* during delivery and infecting their owners through the air [4]. The epidemiological role of dogs in Q fever maintenance, transmission, and spread remains unclear [4].

A study of military army (not police) dogs found seropositivity rates of 34/348 (9.8%) in Southern France, 5/43 (11.6%) in Dakar, Senegal, 1/19 (5.2%) in French Guyana, and 1/12 (8.3%) in Abidjan, Ivory Coast [5]. The seroprevalence of *C. burnetii* in companion dogs has ranged widely worldwide. At the country level, companion dogs’ seroprevalence rates include 5/265 (1.9%) shelters, 7/309 (2.3%) breeding, 10/328 (3.0%) households, 21/321 (6.5%) aboriginal [4], and 44/201 (21.8%) owned dogs [6,7], in the case of Australia. Data on dog serosurveys of *C. burnetii* in several countries have been gathered and presented in Table 1. In Italy, *C. burnetii* was isolated directly from *Rhipicephalus sanguineus* ticks in naturally infected dogs, the same tick species that has been identified throughout Brazil [8]. Although more than 40 tick species are currently capable of carrying *C. burnetii*, their potential for transmission to animals and humans remains unknown [4].

The Military Police—Special Operations Battalion (BOPE)—in Brazil is a group of outstanding state police officers employed in rescue and other high-risk operations, commonly using working dogs in daily routines for drugs, guns, ammunition, money, and human searching and detection [9]. Despite the possibility of acquiring ticks and tick-borne and other zoonotic diseases associated with potential infection and transmission from close and continuous human–animal relationships during such operations, no study to date has focused on working dogs and their owners. Accordingly, the present study aimed to assess and serosurvey *C. burnetii* in police officers and working dogs at the Special Operations Battalion (BOPE-PR) in Curitiba, the eighth-largest city in Brazil with approximately 1.8 million habitants.

**Table 1:** Worldwide results of *Coxiella burnetii* seropositivity in dogs.

Location	Year	Dog Type	Sample	Positivity	Assay	Ref.
Delhi, India	1979	Stray	49	7 (14.3%)	Complement fixation	[10]
California, USA	1980	Stray	316	209 (66.1%)	Microagglutination	[11]
Nova Scotia, Canada	1985	Household	447	0	IFA	[12]
The Netherlands	1987	Household	219	0	ELISA	[13]
Bologna, Italy	1992	Household	802	7 (0.9%)	ELISA	[14]
Kitaoka, Japan	1992	Stray	632	95 (15%)	IFA	[15]
Setúbal, Portugal	1995	Shelter	104	5 (4.8%)	IFA	[16]
Southern Croatia	1995	Stray	51	6 (11.8%)	Complement fixation	[17]
Southeast France	1998	Military	348	34 (9.8%)	IFA	[5]
Dakar, Senegal	1998	Military	43	5 (11.6%)	IFA	[5]
French Guyana	1998	Military	19	1 (5.2%)	IFA	[5]
Abidjan, Ivory Coast	1998	Military	12	1 (8.3%)	IFA	[5]
Iraq	2011	Wild	165	9 (5.5%)	IFA	[18]
Queensland, Australia	2011	Stray	101	22 (21.8%)	IFA	[19]
Sydney, Australia	2016	Breeding	309	7 (2.3%)	IFA	[6]
Sydney, Australia	2016	Household	328	10 (3%)	IFA	[6]
Sydney, Australia	2016	Aboriginal	321	21 (6.5%)	IFA	[6]
Sydney, Australia	2016	Shelter	265	5 (1.9%)	IFA	[6]
Yangzhou, China	2016	Household	136	0	ELISA	[20]
Iran	2016	Household	182	1 (0.6%)	ELISA	[21]
South Korea	2017	Household	1023	30 (2.9%)	IFA/ELISA	[22]
Montenegro	2019	Household	259	3 (1.2%)	ELISA	[23]
Central Italy	2020	Household	516	42 (8.1%)	IFA	[24]
Queensland, Australia	2022	Pig-hunting	104	19 (18.3%)	IFA	[6]
Bangkok, Thailand	2022	Household	570	7 (1.2%)	IFA	[25]
Chile	2022	Rural	358	0	IFA	[26]
Southern Brazil	2024	Quilombo	20	1 (5%)	IFA	[27]
Southern Brazil	2024	Indigenous	406	1 (0.3%)	IFA	DNP

IFA: indirect immunofluorescence assay. DNP: data not published.

## 2. Materials and Methods

This study was approved by the Human Health Ethics Committee (protocol number 3.166.749/2019), Ethics Committee of Animal Use at the Federal University of Paraná (protocol number 040/2023), and Headquarters Command of Military Police in Paraná State (protocol number 21.094.524-5).

Human and dog blood samples were collected on a single day (24 July 2021) during a requested molecular survey of saliva samples for COVID-19; SARS-CoV-2 was not detected in the samples. Policemen were sampled after signing a consent form and completing an epidemiological questionnaire, and blood samples (10 mL) were obtained by cephalic venipuncture conducted by certified nurses. The dogs were sampled after the handlers signed a consent form and completed a dog epidemiological questionnaire. Blood samples (10 mL) were obtained by jugular venipuncture conducted by certified veterinarians. Whole human and dog blood samples were placed in sterile vacuum tubes containing serum separator gel without an anticoagulant. Blood samples were kept at room temperature (25 °C) until visible clots were formed and subsequently centrifuged at 800× *g* for 5 min. The serum samples were kept at −20 °C until use.

For human diagnosis, an in-house immunofluorescence assay (IFA) kit produced in Brazil ("(Ezequiel Dias Foundation, Belo Horizonte, Minas Gerais State, Brazil) containing antigens from the Argentine At12 strain, was used. This kit does not distinguish between phase I and phase II antibodies in its detection. An IgG anti-human fluorescein isothiocyanate (FITC) antibody was used for the tests. For canine diagnosis, a commercial IFA kit (SCIMEDX Corporation, Denville, NJ, USA) produced with Nine Mile antigens was used. This kit distinguishes between phase I and phase II antibodies in its detection. An IgG anti-dog fluorescein isothiocyanate (FITC) antibody was used for the tests. Serologies were performed according to the protocol described by França et al. [28]. Reactions were observed under an Olympus BX53 immunofluorescence microscope (Photonic Solutions Inc., Mississauga, ON, Canada) equipped with a 40× objective lens. For each slide tested, positive and negative controls were prepared using samples from human and canine patients previously diagnosed in our laboratory. The positive samples were subjected to serial dilutions of 1:32, 1:64, 1:128 and so on, until the final titer was reached. Previous studies in dogs and the Center for Disease Control and Prevention (CDC) manual for human diagnosis were used to define the cut-off. The cut-off point for dogs was set at 1:32, and the cut-off point for humans was set at 1:64.

Fisher’s exact test was used to compare the prevalence of *C. burnetti* seropositivity among the binary variables. For variables with three or more possible answers, a chi-squared test was used. The significance level was set at *p* = 0.05. All tests were performed using SAS Studio 3.81 (SAS Institute Inc., Cary, NC, USA).

## 3. Results

Overall, 1/18 (5.5%) police officers tested by the in-house IFA and 9/30 (30.0%; CI 95% 16.66–47.88) dogs tested by a commercial IFA Kit were seropositive to anti-*C. burnetii* IgG antibodies. This is the highest prevalence of Q fever seropositivity among military dogs worldwide to date. The human seropositive sample presented a phase II antibody titer of 128 and was from a male, 38 years old, with no history of pneumonia or other respiratory diseases, and a handler during the operations of a seronegative dog. Of the nine seropositive dogs, six had antibody titers for both phases, two had only anti-phase I antibodies, and one had only anti-phase II antibodies. Seven dogs had titers of 64 and two had titers of 32. Variables related to breed, sex, age, work specialty and method of acquisition were statistically compared with seropositivity, and the respective results are presented in Table 2.

The nine seropositive dogs were from the Malinois Shepherd and Bloodhound breeds, with the age and method of acquisition not affecting the outcome. The results showed higher seropositivity in search dogs, which work in a variety of operations, including in forest areas, and in female dogs, the latter with a statistically significant association, being 7.6 times more likely to be seropositive than male dogs.

## 4. Discussion

The seropositivity presented in this study may provide important data on canine infection by *C. burnetii,* particularly regarding why working dogs may be more vulnerable to infection than domestic dogs, as was also observed in military dogs in the French Army [5] when compared to other dog categories worldwide (Table 1). As higher seropositivity rates in dogs are reportedly uncommon when compared with humans [1], the dogs examined in this study may have been infected by ticks during operations or at the kennels in the BOPE headquarters.

Q fever seroprevalence in domestic and shelter dogs has varied from 0 to 8.1%, whereas that in working dogs ranged from 5.2% to 18.3% (Table 1). Likewise, our results indicated that dog occupations were the likely higher associated risk factor for *C. burnetii*, as military police dogs routinely participate in search operations in forest, woody, and natural areas. A study in Australia showed that Aboriginal dogs were more likely to be infected with *C. burnetii* than domestic and shelter dogs because of higher contact with bushland and wildlife [6]. As all the dogs in this study had a history of tick infestation, contact with the natural environment and wildlife may have increased infection risk of *C. burnetii* when compared to domestic dogs living in urban settings. Due to the limited number of studies to date, the prevalence of *C. burnetii* infection among dogs in the Americas remains uncertain.

Although a *C. burnetii* infection may cause abortion, impact dog health, and impair working dog breeding, dogs in the Brazilian police battalion are mostly neutered or spayed and used for operational and not breeding purposes. Nonetheless, neutered dogs have an increased risk of being seropositive compared to intact dogs, which may occur because of the increased age of neutered dogs and greater lifetime exposure than younger dogs [7]. However, this may not be the case in the present study, in which the working dogs were all adults and seropositivity was not associated with age. In addition, dogs living within the same household have shown a higher risk of seropositivity due to shared exposure to environmental infection sources [7], as observed in working dogs in this study, who shared operational incursions at work, and rested at the police battalion kennels. In another study, dogs living in rural and agricultural regions also presented an increased risk of seropositivity, perhaps because of contaminated soil and dust from livestock farming.

In addition, marsupials in Zambia [29] and wild pigs in Australia [7] have shown *C. burnetii* seropositivity due to wildlife and nature exposure. Police dogs working in natural and rural areas that are part of the different ecosystems (Atlantic and Rain Forests, Savanah, and Mangrove swamps) of Paraná state, including the Argentinian and Paraguayan borders, should be considered vulnerable. Not surprisingly, *C. burnetii* DNA was found in 15/150 (10.0%) dogs in Zambia [29] and in 113/276 (41.0%) ticks collected from 90 dogs in South Africa, including *R. sanguineus*, *Haemaphysalis elliptica* and *Amblyomma hebraeum* tick species [30], which are also found in Brazil. Although tick-borne transmission is a major infection factor in working dogs, ticks were not found at the time of the survey due to periodic preventive programs with recently applied tick repellents.

From the One Health approach to Q fever, this study highly recommends a concomitant serosurvey of police officers and working dogs, along with environmental surveys. In such a scenario of human–animal daily sharing exposure at work, infection in dogs may impact human transmission and vice versa, as already observed for other zoonoses in vulnerable populations, such as homeless, incarcerated, indigenous, and traditional island populations [31].

Thus, the seropositive policeman in this study may serve as a warning of the airborne exposure. The working dogs sampled herein were neutered and unlikely to eliminate the agent; thus, they were no longer considered sources of infection. As a limitation, the risks of exposure of seropositive police officer in his personal activities have not been assessed, and for this reason, any evaluation cannot be carried out. Regardless, this case report should be interpreted as an alarming One Health professional risk and workers’ health concern for police K-9 Units worldwide.

Seroprevalence in animals has usually been associated with the presence of nearby livestock farms or animal slaughterhouses. This is not the case in Curitiba, the city where the study was carried out. There are no slaughterhouses or livestock farms within 30 to 50 km of where the dogs live, and because it is a wooded area, it is not often windy, which would make it easier for the spores to spread. Another recurring explanation is food-borne infections, mainly due to the consumption of raw meat [32], but these animals are fed exclusively with dog food, according to the officers. Work operations are sometimes carried out in natural areas and contact with wild species and their ticks may explain the exposure. Among the mammals belonging to the local biodiversity are bats, skunks, and armadillos, and *C. burnetii* infections have already been described in bats and marsupials in Brazil [33]. In the operations, the animals are prevented from having physical contact with other species, but the role of wild animals in dispersing the bacteria in the environment is still unknown. In addition, it has been observed that the control of ectoparasites is inefficient, since the purchase of antiparasitic drugs is made through public financial transfers, which does not occur periodically. Contact with ticks may explain the high seropositivity of the group [34]. For most researchers, the potential for transmission from dogs to humans is low [35]. In addition to cattle, dogs and cats that are in close contact with humans are important potential reservoirs of *C. burnetii* during urban outbreaks of Q fever. Some cases of human infection have already been described as having dogs and cats as sources of infection; however, several pets have been found to be positive in investigations of human clusters, without these animals being incriminated in the transmission to humans [33].

Despite the reported transmission through contact with postpartum vaginal fluids, the role of dogs in the human Q fever cycle remains uncertain because this infection route has rarely been associated with human infections [36]. A single human case report has been associated with dog infection to date, during a Q fever outbreak with pneumonia that impacted three family members twelve days after exposure to an infected whelping dog, whose entire litter, sadly, did not survive [37]. Thus, further studies should be conducted on the reproductive kennels of the seropositive dog breeds found by this study, thus ruling out *C. burnetii* contact prior to admission to the Police K-9 Units.

Although a 128-cut-off point has been established to determine acute illness in people with fever [38], the same titer of the seropositive policeman examined in this study was designed as a serosurvey of clinically healthy individuals. However, even in asymptomatic cases, *C. burnetii* may remain in the body for years and cause serious complications, such as endocarditis and hepatitis [39,40,41]. The two dogs seropositive only for anti-phase I antibodies may have shown an infection of more than six months, where phase II antibodies may decline and end, while phase I antibodies may increase and remain stable, with *C. burnetii* persisting over time [41].

Finally, this study found that female dogs were statistically more seropositive than male dogs, and a trend of dogs specializing in search operations being more exposed than others. Dogs bred and used from a young age in operations had a tendency of higher seropositivity than dogs acquired through purchase or donation. As dogs become at risk of infection at the time of delivery, or in cases of miscarriage and during lifetime exposure [7,38], the results of the study have corroborated higher exposure in females, search dogs, and long-exposed working dogs.

One limitation of the present study was the relatively low number of samples, which was caused by the one single sampling. In addition, part of the officers and dogs were out due to statewide duties. Further studies should be conducted with a higher number of samples and in different populations to fully establish the exact impact of *C. burnetti* in police officers and working dogs.

Human and dog blood samples were collected on a single day (24 July 2021) during a requested molecular survey of saliva samples for COVID-19; SARS-CoV-2 was not detected in the samples.

However, serological testing may not be a reliable method for determining whether specific animals are potential sources of *C. burnetii* transmission to humans. In this study, dog seropositivity to *C. burnetii* may only indicate previous exposure with no precise time of exposure or whether the exposure resulted in a clinical or subclinical disease.

## 5. Conclusions

This study was the first to undertake a concomitant serosurvey of police officers and working dogs, with 1/18 (5.5%) police officers and 9/30 (30.0%) dogs seropositive for anti-*C. burnetii* IgG antibodies, which is the highest prevalence of Q fever seropositivity among military dogs worldwide to date. Despite the low sampling rate, a statistically significant association was found between seropositivity and female dogs (*p* = 0.0492). The results of this study should be considered a warning for cross-exposure and transmission of *Coxiella burnetii* among Police K-9 Units in Brazil and worldwide.

## Figures and Tables

**Table 2:** Demographics data of working dogs from the Special Operations Battalion of Curitiba, Brazil (2023) and their respective seropositivity for Q fever.

Variables	*C. burnetii* Positive	*C. burnetii* Negative	OR (95% CI)	*p* Value	Total Population
Breed				0.8389	
German Shepherd	0 (0.0%)	4 (100.0%)	1.0 (ref)		4
Malinois Shepherd	8 (36.4%)	14 (63.6%)	0.19 (0.00–3.97)		22
Bloodhound	1 (33.3%)	2 (66.7%)	0.18 (0.00–6.47)		3
Holland Shepherd	0 (0.0%)	1 (100.0%)	0.33 (0.00–25.4)		1
Age				0.6814	
Adults	7 (33.3%)	14 (66.7%)	1.0 (ref)		21
Seniors	2 (22.2%)	7 (77.8%)	1.75 (0.28–10.7)		9
Sex				0.0492 *	
Female	4 (66.7%)	2 (33.3%)	1.0 (ref)		6
Male	5 (20.8%)	19 (79.2%)	7.6 (1.06–54.1)		24
Specialty				0.1618	
Narcotics	3 (17.6%)	14 (82.4%)	1.0 (ref)		17
Explosives	1 (25.0%)	3 (75.0%)	0.64 (0.04–8.51)		4
Search	4 (66.7%)	2 (33.3%)	0.10 (0.01–0.88)		6
RPC	1 (33.3%)	2 (66.7%)	0.42 (0.02–6.40)		3
Acquisition				0.7256	
Donation	4 (26.7%)	11 (73.3%)	1.09 (0.15–7.80)		15
Purchase	2 (25.0%)	6 (75.0%)	0.64 (0.04–8.51)		8
Creation	3 (42.9%)	4 (51.1%)	0.48 (0.07–3.19)		7

* Significant *p* Value.

## Data Availability

Data are contained within the article.
